# Generative Artificial Intelligence: Enhancing Patient Education in Cardiovascular Imaging

**DOI:** 10.1093/bjro/tzae018

**Published:** 2024-07-17

**Authors:** Ahmed Marey, Abdelrahman M Saad, Benjamin D Killeen, Catalina Gomez, Mariia Tregubova, Mathias Unberath, Muhammad Umair

**Affiliations:** Alexandria University Faculty of Medicine, Alexandria, 21521, Egypt; Alexandria University Faculty of Medicine, Alexandria, 21521, Egypt; Department of Computer Science, Laboratory for Computational Sensing and Robotics, Johns Hopkins University, Baltimore, MD, 21218, United States; Department of Radiology, Amosov National Institute of Cardiovascular Surgery, Kyiv, 02000, Ukraine; Department of Computer Science, Laboratory for Computational Sensing and Robotics, Johns Hopkins University, Baltimore, MD, 21218, United States; Russell H. Morgan Department of Radiology and Radiological Sciences, The Johns Hopkins Hospital, Baltimore, MD, 21205, United States

**Keywords:** Cardiovascular diseases, Patient education, Generative artificial intelligence, resource-limited settings

## Abstract

Cardiovascular disease (CVD) is a major cause of mortality worldwide, especially in resource-limited countries with limited access to healthcare resources. Early detection and accurate imaging are vital for managing CVD, emphasizing the significance of patient education. Generative artificial intelligence (AI), including algorithms to synthesize text, speech, images, and combinations thereof given a specific scenario or prompt, offers promising solutions for enhancing patient education. By combining vision and language models, generative AI enables personalized multimedia content generation through natural language interactions, benefiting patient education in cardiovascular imaging. Simulations, chat-based interactions, and voice-based interfaces can enhance accessibility, especially in resource-limited settings. Despite its potential benefits, implementing generative AI in resource-limited countries faces challenges like data quality, infrastructure limitations, and ethical considerations. Addressing these issues is crucial for successful adoption. Ethical challenges related to data privacy and accuracy must also be overcome to ensure better patient understanding, treatment adherence, and improved healthcare outcomes. Continued research, innovation, and collaboration in generative AI have the potential to revolutionize patient education. This can empower patients to make informed decisions about their cardiovascular health, ultimately improving healthcare outcomes in resource-limited settings.

## Introduction

Cardiovascular disease (CVD) is a leading cause of morbidity and mortality worldwide.^1^ According to the World Health Organization, an estimated 17.9 million people died from CVDs in 2019, representing 32% of all global deaths.^2^ Moreover, the burden of CVD is particularly high in resource-limited countries, where access to healthcare and resources for prevention, diagnosis, and treatment is often limited.^3^ In fact, although incidence rates across European Society of Cardiology member countries have declined by ∼40%, in the last 30 years, they remain twice as high in middle-income compared with high-income countries.^4^ Furthermore, patients in resource-limited countries are particularly vulnerable to its devastating effects due to high prevalence rates and low awareness of prevention strategies.^3^ Over three-quarters of CVD deaths take place in low- and middle-income countries.^2^

Early detection and treatment of CVD are critical for improving patient outcomes, and accurate imaging and diagnosis are key components of this process.^5^ Cardiovascular imaging techniques have become an essential part of patient management. In addition, patient education and understanding of the information gained from imaging is crucial for effecting preventative behaviour and treatment.^5^

However, patient education regarding cardiovascular imaging, especially in resource-limited countries, remains a challenge. It often relies on traditional methods, such as printed materials, verbal instructions, and face-to-face interactions with healthcare providers. Nevertheless, these methods may be limited by factors such as low health literacy, language barriers, and limited access to trained healthcare professionals. As a result, there is a need for innovative and effective approaches to patient education in these settings.^6–8^

Recently, generative artificial intelligence (AI) has emerged as a promising tool for improving patient education and subsequent patient outcomes in a variety of healthcare settings, including resource-limited countries.^9^^,^^10^ Generative AI refers to a subset of AI that uses algorithms to generate new data or content, such as images, audio, and video files, that are similar to existing data.^11^^,^^12^ Among these techniques are conditional generative AI techniques, that can generate specific content based on a conditioning input signal, such as a text or voice prompt. In healthcare settings, generative AI has been used to improve patient education and outcomes in a variety of ways.^13^^,^^14^ For example, generative AI has been used to create personalized educational materials for patients with chronic diseases such as diabetes.^15^

There are several specific AI technologies or applications that have been successful in improving patient education in other areas of healthcare. Namely, chatbots have been used to provide patients with personalized health information and support.^15^^,^^16^ Given these advancements, this article will explore the potential role of generative AI in enhancing patient education regarding cardiovascular imaging in resource-limited countries. By leveraging generative AI, healthcare providers can deliver tailored educational content to patients, thereby promoting awareness of CVD prevention strategies, facilitating early detection of CVDs, and ultimately improving patient outcomes.

This exploration is critical not only for enhancing patient education but also for potentially saving valuable time for healthcare providers. The efficient dissemination of educational materials through generative AI platforms has the potential to allow healthcare providers to allocate resources more effectively and focus on delivering quality care to patients. Failure to adequately educate patients about cardiovascular imaging procedures can have significant consequences, including delays in diagnosis, suboptimal treatment outcomes, and increased healthcare costs. Therefore, by addressing these gaps in patient education through the innovative application of generative AI, this article seeks to contribute to efforts aimed at reducing the burden of CVD in resource-limited countries and promoting health equity on a global scale.

## An overview and introduction to generative models

Machine learning models typically fall into 2 categories: discriminative and generative. Discriminative models focus on predicting characteristics based on observed data (P(Y│X)); in medical applications, the observed data may consist of a medical image while the output characteristics include anatomical structures, disease presence, or pathology.^17^ While generative models predict the likelihood of observing data given prior information (P(X│Y)). Several types of generative models have been proposed, each of which accomplishes this goal with varying levels of success.

### Vision-based generative models

**Figure 1.**
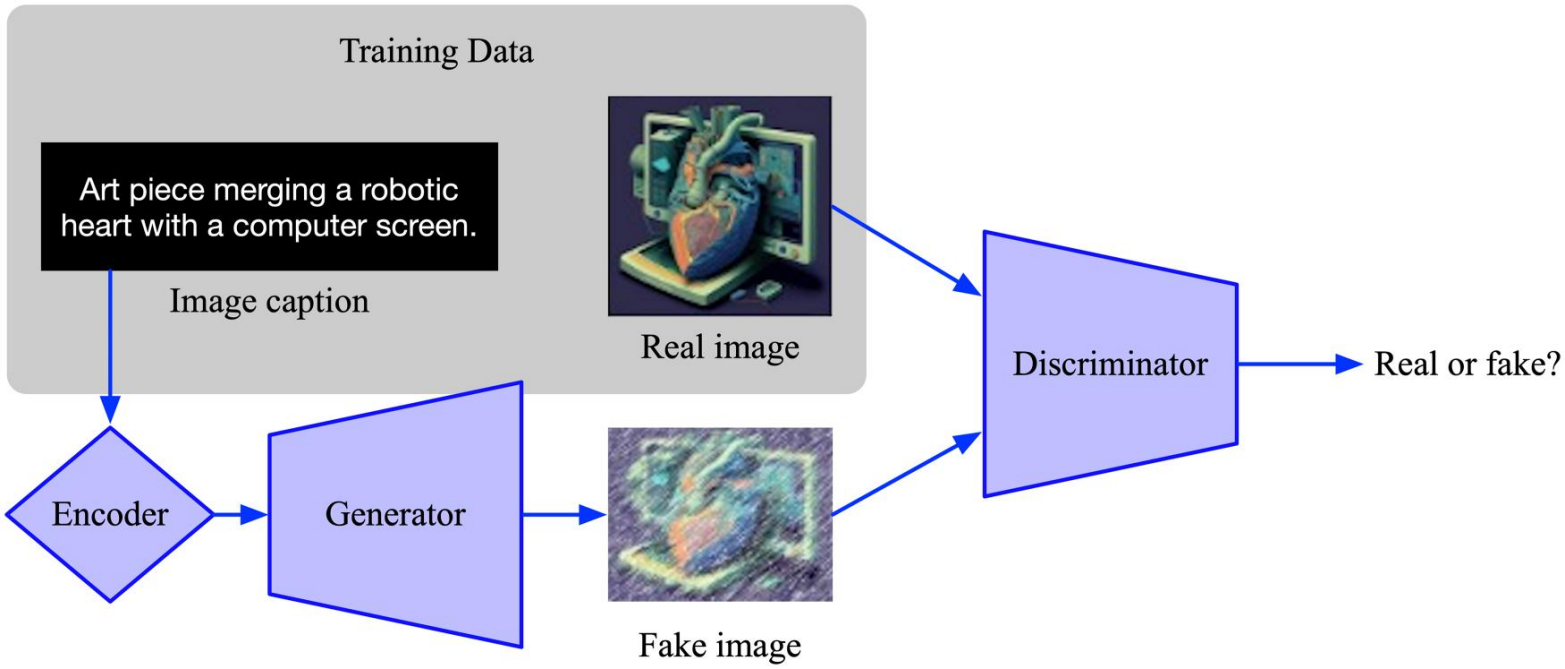
Illustration of the training process for a text-to-image generative adversarial network model. Text input undergoes encoding and is fed into a generator, producing an image. The discriminator evaluates the authenticity of the generated image in an adversarial training loop, optimizing both discriminator and generator performance iteratively to enhance image quality.

Early attempts at vision-based generative models faced challenges in image realism. Autoencoders, initially used for pre-training discriminative models, could generate new images but often produced blurry or unrealistic results.^18^ Variational autoencoders (VAEs) addressed this by structuring the latent space, improving image generation and anomaly detection.^19^ Generative adversarial networks (GANs), introduced by Goodfellow in 2014, revolutionized generative models, producing realistic images.^20^ By learning to fool the discriminator, the generator network gradually produces images that are increasingly similar to the training data. Eventually, the generator network can create synthetic samples that are difficult for humans to distinguish from real samples.^21^ Over time, GAN variants such as Conditional GANs and Progressive GANs, Info GANs, Cycle GANs, and Dual GANs emerged, expanding applications to text-to-image models.^22^Figure 1 represents the training process for a text-to-image generative adversarial network model.

**Figure 2.**
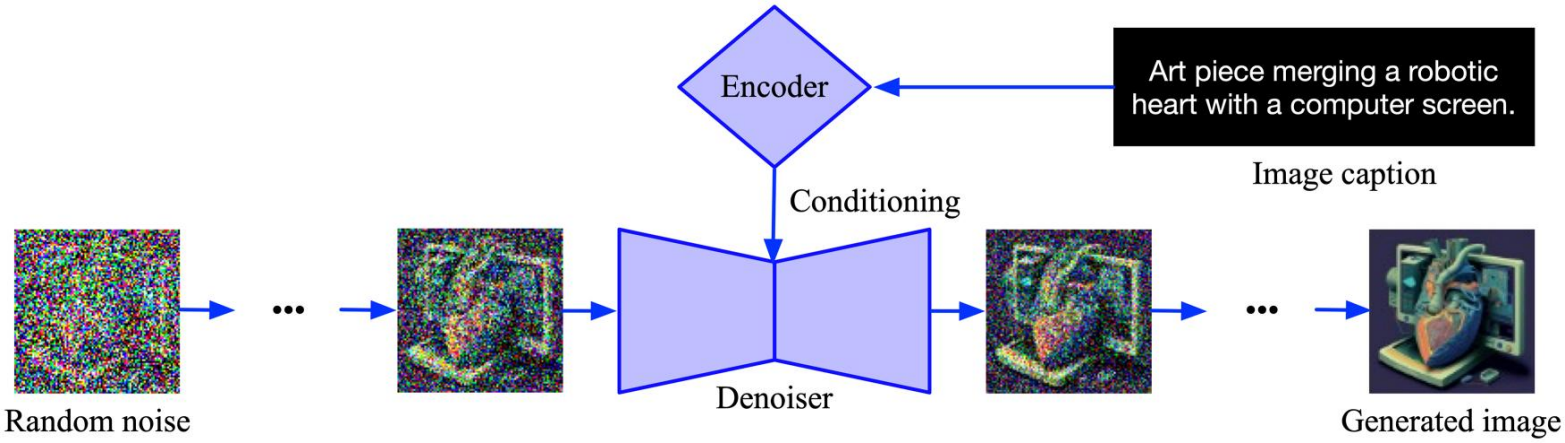
Illustration of the training process for a diffusion model. Training of a diffusion model involves feeding encoded text/data and random noise into the model. Through iterative processing, the model gradually transforms this input, ultimately generating the final image output.

Despite GANs’ dominance, denoising diffusion models and Vector Quantized-VAEs (VQ-VAEs) have gained prominence. VQ-VAEs, solved the “posterior collapse” issue, where the decoder essentially ignores the latent code based on which it should generate an image. By using discrete, rather than continuous, latent codes and learning the prior distribution, VQ-VAEs produce much more realistic images than VAEs.^23^ Diffusion models offer high-quality, high-resolution images. Rather than generate an image with a single pass of a neural network, diffusion models frame the image generation process as a denoising problem, in which multiple passes gradually convert a noisy image into a realistic one.^24^Figure 2 overviews the generative process for diffusion models. Although diffusion models still require significant computational resources to both train and deploy compared to GANs, their advantages in terms of image realism have made them the frontrunner in generative AI going forward.^25^

### Language-based generative models

Language models (LLMs) for natural language processing (NLP) assign probabilities to sequences of words. Neural networks have demonstrated improved performance in language understanding tasks with discriminative models and extended capabilities to generate open-ended text in tasks such as summarization, machine translation, and dialogue and code generation. Early sequence-to-sequence which used recurrent neural networks faced limitations in handling global dependencies.^26^ Transformer architectures, relying on self-attention mechanisms, addressed these challenges and significantly improved language understanding tasks.^27^

Generative pre-training transformers (GPT) models, introduced by OpenAI, leverage transformer architecture for language understanding.^27^ The working hypothesis of these models is that by forcing the language model to predict the next word, the model most discover and learn useful patterns and correlations in the input data, especially when trained on large amounts of text. The recent evolution of these models has demonstrated that LLMs can increase their capability as the size of the training data and parameter space increases.^28^

Even though GPT-3 was trained on abundant data from the internet and can produce human-like text, its output can exhibit unintended behaviours or biases given the open-ended power of LLM. The popular ChatGPT model released last year achieved an improvement in alignment with human expectations and desirable values, an important feature for potential applications as dialogue systems or personal assistants. This approach is based on the Instruct GPT model that incorporates human feedback.^29^ Human preferences are collected from labellers and used to train a model that predicts which model output would be preferred by labellers, which is further used as the reward signal to fine-tune the initial supervised model.

### Multimodal models

On their own, LLMs and generative vision models are promising tools, but taken together they represent significant potential by providing casual users with a natural interface for controlling generation of multimedia content.^30^ State-of-the-art models can generate realistic image sequences, audio files, and 3D models based on text descriptions of the desired output, and vice versa. Users can interact with these models as they would another human being, requesting changes and refining output in a “chat” until the desired effect is achieved. As these methods advance, their applications in engaging and educating patients must be carefully considered so as to broaden access to and understanding of crucial health information.

## Potential generative AI applications in patient education

Generative AI has the potential to transform patient education regarding cardiovascular imaging in resource-limited countries. By leveraging machine learning algorithms and other advanced technologies, generative AI can create new opportunities for personalized, accessible, and engaging patient education materials (Table 1). Here are some ways in which generative AI can be used to improve patient education in these settings:

**Table 1.** Potential applications of gen-AI in patient education.

Application	Description
Multimedia content generation	Creation of engaging videos, animations, and infographics to explain complex medical concepts such as cardiovascular imaging procedures.
Text-to-image creation	Conversion of written descriptions of medical procedures into visually informative images or illustrations for better understanding.
Simulation	Development of virtual reality simulations for patients to experience medical procedures in a safe and controlled environment.
Predictive analytics	Utilizing patient data to identify trends and patterns for targeted education initiatives.
Chatbots	Creation of chatbots to provide personalized health information and support to patients in real-time.
Virtual assistants	Development of virtual assistants to guide patients through imaging procedures and provide step-by-step instructions.

### Multimedia content generation

Generative AI can be used to create multimedia content such as videos, animations, and infographics for patients that are engaging and informative.^31–34^ In particular, AI algorithms can create interactive videos or animations that demonstrate how the cardiovascular system works or how imaging techniques are used to diagnose heart diseases. Similarly, educational videos can be created on patient preparation before a cardiovascular imaging exam or what to expect during a cardiovascular imaging examination. For example, AI models can generate animations that illustrate the process of echocardiography or CT scanning, making it easier for patients to understand and remember.^34^

In addition, generative AI can be employed to create visually engaging and informative content by transforming natural descriptions into images or videos. By conditioning generation on text, AI systems can generate detailed visuals that accurately represent the information desired, enhancing the patient education experience.

#### Text-to-image creation

Generative AI algorithms can convert written descriptions of cardiovascular imaging procedures or concepts into accurate, informative images or illustrations.^31^^,^^35^ This can help patients better understand the information by presenting it in a more visually appealing and digestible format. For instance, AI can generate an image illustrating the steps of an echocardiogram based on a textual description, making it easier for patients to comprehend the procedure.

#### Video creation

Generative AI can also be used to create educational videos based on textual content. By converting text descriptions into animations or videos, AI can provide a more interactive and engaging learning experience for patients.^36^^,^^33^ In particular, AI may create a video demonstrating the process of a CT coronary angiogram, helping patients understand the procedure and its purpose in diagnosing heart disease.

#### Virtual assistants

Generative AI can be used to create virtual assistants that can guide patients through cardiovascular imaging procedures.^37^ Namely, the AI system can create a virtual assistant that provides patients with step-by-step instructions on how to prepare for an imaging procedure and what to expect during the procedure.^38^ Virtual assistants can help reduce anxiety and address other concerns related to medical procedures, contributing to a more positive patient experience and increased patient confidence. In addition, virtual assistants can also be used to assist visually impaired learners.^39^^,^^40^

### Personalization

Generative AI can create personalized education programs tailored to individual patient's learning styles and preferences when it comes to understanding cardiovascular imaging procedures and their results.^41–43^ For example, the AI system can analyse patient data to identify areas of strength and weakness, as well as their specific cardiovascular health conditions, and then adapt the education program accordingly.^44^^,^^45^ This can help patients learn at their own pace and in a way that is most effective for them, facilitating a better grasp of cardiovascular imaging concepts, such as the types of imaging techniques used, the purpose of each procedure, and how to interpret the results. For example, a generative AI-enabled chatbot can be created that answers patient’s questions about different imaging procedures and provides guidance on what they are going through.^46^ A patient who is scheduled for a procedure such as CT coronary angiography, which requires prior dietary and, in some cases, medical preparation, may receive text messages with a reminder of the upcoming examination and the need to complete the tasks. Table 2 provides examples of studies that assessed the potential role of chatbots in advancing patient education about medical imaging.

**Table 2.** Studies assessing the role of chatbots in advancing patient education on medical imaging.

Study ID	Year	Study design	AI model used	Aim of the study	Methods	Results	Implications for practice	Conclusion
Kuckelman 2024^58^	2024	Case Study	Bing Chatbot	To evaluate the performance of the Bing Chatbot in providing responses to questions related to CT abdomen, MRI spine, and bone biopsy.	Tested Bing Chatbot responses to 10 questions each for CT abdomen, MRI spine, and bone biopsy. Used 3 different chatbot settings.	93% of reviews were entirely correct, 7% mostly correct. 65% of responses were complete, 35% mostly complete. No significant difference based on settings or exam types. Readability level: eighth-grade.	Bing Chatbot provided accurate responses, demonstrating reliability and potential for enhancing patient education in radiology.	The Bing Chatbot demonstrated high accuracy and completeness in providing information on common radiologic exams. It could be integrated into patient portals for various purposes, including exam preparation and results interpretation.
Kuckelman 2024^59^	2024	Feasibility Study	ChatGPT-4	To investigate the feasibility of using ChatGPT-4 to generate layperson summaries for musculoskeletal radiology reports.	Obtained 60 musculoskeletal radiology reports (20 MR shoulder, 20 MR knee, 20 MR lumbar spine) from PACS. Reports were deidentified and submitted to ChatGPT-4 to generate layperson summaries. Summaries evaluated by 3 independent readers for completeness and accuracy.	Ratings from 1 to 3 were given (1: Worst, 3: Best). Mean accuracy ratings: 2.58, 2.71, and 2.77. Mean completeness ratings: 2.87, 2.73, and 2.87. Low inter-reader agreement for accuracy (kappa 0.33) and completeness (kappa 0.29).	Overall, AI-generated layperson summaries were rated highly accurate and complete, with only a small minority likely to be confusing or inaccurate. Demonstrates potential for AI to automate patient-friendly summaries for musculoskeletal MR imaging.	The study illustrates the potential of leveraging generative AI like ChatGPT-4 to automate patient-friendly summaries for musculoskeletal MR imaging, with high ratings for accuracy and completeness.
Rahsepar 2023^60^	2023	Comparative study	ChatGPT-3.5; Goggle Bard; Bing; Google search engine	To compare the accuracy and consistency of responses provided by ChatGPT-3.5, Google Bard, Bing, and Google search engine for questions related to lung cancer prevention, screening, and radiology terminology.	Created 40 questions related to lung cancer prevention, screening, and radiology terminology. Presented questions to ChatGPT-3.5, Google Bard, Bing, and Google search engines. Each answer reviewed by 2 radiologists for accuracy. Consistency evaluated among answers.	ChatGPT-3.5: 70.8% correct, 11.7% partially correct, 17.5% incorrect. Google Bard: 51.7% correct, 9.2% partially correct, 20% incorrect. Bing: 61.7% correct, 10.8% partially correct, 27.5% incorrect. Google search engine: 55% correct, 22.5% partially correct, 22.5% incorrect.	ChatGPT-3.5 more likely to provide correct or partially correct answers than Google Bard. ChatGPT-3.5 and Google search engine more consistent than Google Bard.	ChatGPT-3.5 had higher accuracy compared to other tools, but none answered all questions correctly and with 100% consistency.
Khurana 2023^61^	2023	Case study	ChatGPT	To investigate the effectiveness of ChatGPT in generating radiology reports based on the specificity and clarity of prompts provided by radiologists.	Radiologists used ChatGPT to generate radiology reports based on prompts. Two examples provided. In the first example, the prompt lacked specificity, resulting in a report with deficiencies and no differential diagnosis. In the second example, a precise prompt led to a comprehensive report including location, extent, size, shape, radiographic appearance, clinical implications, differential diagnosis, and recommendations.	ChatGPT's effectiveness in generating radiology reports depends on the specificity and clarity of the prompts provided by radiologists. Precise prompts lead to more comprehensive and accurate reports.	Radiologists must understand the importance of providing precise prompts to ChatGPT for generating accurate and comprehensive radiology reports.	ChatGPT can generate decent automated radiology reports, but its effectiveness relies heavily on the clarity and specificity of the prompts provided.
Rogasch 2023^62^	2023	Evaluation Study	ChatGPT	To assess the appropriateness and usefulness of ChatGPT in explaining PET/CT reports and answering follow-up questions.	Thirteen questions about [18F]FDG PET/CT submitted to ChatGPT. ChatGPT asked to explain 6 PET/CT reports and answer 6 follow-up questions. Responses rated “appropriate” or “useful” if adequate by nuclear medicine staff standards. Inconsistency assessed by regenerating responses.	Responses rated “appropriate” for 92% of tasks and “useful” for 96%. Considerable inconsistencies found in 16% of tasks upon regeneration. Responses to 83% of sensitive questions rated “empathetic.”	ChatGPT may adequately substitute for advice given by nuclear medicine staff to patients in investigated settings. Improvement in consistency would enhance reliability.	ChatGPT demonstrates potential to provide useful and appropriate responses to patient questions about [18F]FDG PET/CT, but consistency improvements are necessary to enhance reliability.
Wagner 2024^63^	2023	Accuracy assessment	ChatGPT-3	To evaluate the accuracy of responses provided by ChatGPT-3 to radiologists' questions.	Eighty-eight questions from radiologists' daily routine submitted to ChatGPT-3. Responses assessed for correctness by cross-checking with PubMed-listed references. References provided by ChatGPT-3 evaluated for authenticity.	67% of responses to radiological questions correct; 33% contained errors. Out of 343 references provided, only 36.2% available through internet search, with 63.8% appearing to be hallucinated by ChatGPT-3. Only 37.9% of identified references provided enough background to answer questions correctly.	Caution advised when using ChatGPT-3 to retrieve radiological information due to errors in responses and lack of authentic references.	ChatGPT-3 provided correct responses to only about two-thirds of radiological questions in this pilot study. Majority of provided references not found or lacked correct information. This has implications in accuracy and utility of patient education generated using GPT.
McCarthy 2023^64^	2023	Comparative study	ChatGPT	To compare the readability, factual correctness, and suitability for patient education between ChatGPT-generated content and content from a patient education website.	Content from the Society of Interventional Radiology Patient Center website categorized and organized into questions. Questions entered into ChatGPT platform, output analysed for word and sentence counts, readability, factual correctness, and suitability for patient education using PEMAT-P tool.	ChatGPT output longer and more difficult to read on 4 of 5 readability scales compared to website content. ChatGPT incorrect for 11.5% of questions. ChatGPT content scored lower than website material using PEMAT-P tool. Both website and ChatGPT content significantly above recommended grade level for patient education.	Providers should be aware of limitations of ChatGPT in producing patient educational content. Opportunities exist to improve large language models for delivery of patient education.	ChatGPT may produce incomplete or inaccurate patient educational content. Providers should be cautious in relying on ChatGPT for patient education. There is potential to fine-tune existing large language models for better patient education delivery.
Patil 2024^65^	2024	Comparative study	ChatGPT; Bard	To compare the performance of ChatGPT and Bard in providing information about imaging scenarios regarding risks, benefits, and alternatives.	Fourteen imaging-related scenarios used, including factors like use of contrast, renal disease presence, and pregnancy. Three prompts for risks, benefits, and alternatives input into ChatGPT and Bard. Responses graded by 2 reviewers using Likert scale. Prompt variability and chatbot context dependency assessed.	ChatGPT outperformed Bard in accurately responding to prompts. Substantial agreement between reviewers for both chatbots. Response length similar between ChatGPT and Bard. ChatGPT had longer response time.	ChatGPT may be a better resource for outlining risks, benefits, and alternatives in imaging scenarios compared to Bard. Both chatbots have limitations in providing detailed scientific reasoning and patient-specific information.	ChatGPT performs better than Bard in providing information about imaging scenarios. However, limitations exist for both chatbots in delivering detailed scientific reasoning and patient-specific information.

By analysing patient data, generative AI can also provide tailored information on cardiovascular health and imaging, helping patients understand complex concepts and make informed decisions.^32^^,^^47^ This personalized approach can lead to improved patient outcomes and satisfaction, as well as more effective communication between patients and healthcare providers. Ethical considerations towards patient privacy and confidentiality play a crucial role while developing such tools. It is essential to strike a balance between personalization and respecting patient's subjective preferences regarding their privacy and confidentiality.

Notably, GPT-4 stood out in personalized patient education. Unlike static educational materials, such as pamphlets or videos, GPT-4 can engage in real-time conversations with patients, responding to their specific questions and concerns.^48^ For example, an interactive tool can be designed to inform patients about their cardiac CT or MRI with what to expect before, during, and after the appointment, how to prepare, and what to expect from the dedicated cardiac imaging planned. This customization ensures that the information is directly relevant to the patient, which can help improve patient engagement and understanding ultimately empowering them to make informed decisions about their cardiovascular health.

### Accessibility

Generative AI can create content that is accessible to patients with different levels of literacy and language proficiency, enhancing their understanding of cardiovascular imaging procedures and results. For example, generative AI can create videos with captions in multiple languages or with narration in various languages, ensuring that patients from diverse linguistic backgrounds can access and comprehend the information.^49^^,^^50^ Additionally, chatbots were created that can provide patients with information about cardiovascular disease and imaging techniques in their mother languages and consider their respective levels of health literacy.^16^^,^^51^

Generative AI can also be used to create accessible content for patients with disabilities, promoting inclusivity in cardiovascular imaging education.^52^ In particular, the AI system can create videos with closed captions or audio descriptions for patients with visual impairments, helping them understand essential aspects of cardiovascular imaging procedures. In addition, AI algorithms can generate text-to-speech or sign language videos to accommodate patients with hearing impairments,^53^ ensuring that they have equal access to vital educational content.

Furthermore, generative AI can also adapt content based on a patient’s cultural background or any other factors that may influence their understanding of and engagement with the material.^54^ By considering cultural nuances and sensitivities, AI-generated educational content can be made more relevant and relatable to patients, empowering them to make informed decisions about their cardiovascular health and imaging options.

For example, GPT4, which is publicly available, can also help bridge the communication gap between cardiovascular imaging personnel and patients.^55^ It can serve as an intermediary, translating complex medical jargon into layman’s terms and facilitating a better understanding of cardiovascular imaging procedures. Additionally, GPT-4 can potentially support cardiovascular radiologists and imaging technologists in understanding and addressing the unique needs of patients from diverse cultural backgrounds or with different levels of health literacy. This improved communication can lead to more informed decision-making and increased patient satisfaction.

### Simulation

Generative AI can create simulations that allow patients to explore the cardiovascular system or experience medical procedures in a safe and controlled environment.^10^ For example, the AI system can create a virtual reality simulation of a heart catheterization procedure, allowing patients to learn about the procedure and experience it in a realistic way. This approach can help to reduce anxiety and increase patient confidence during imaging procedures. Additionally, generative AI can create simulations and virtual training programs that provide simulated patient experience undergoing cardiovascular imaging examination. These programs can be accessed remotely, enabling healthcare professionals in rural areas to access training and expertise.^56^^,^^57^

### Voice-based interfaces

Generative AI can be used to create voice-based interfaces that recognize different languages in middle- and low-income countries that allow patients to interact with educational content using voice commands.^66^^,^^67^ For example, the AI system can create a voice assistant that allows patients to ask questions about cardiovascular imaging, the procedure, preparation for it, as well as interpretation of the results, and receive answers in a conversational format. Voice-based interfaces can be particularly beneficial for patients with low literacy levels or those who might have difficulty reading text-based materials, enabling them to access crucial health information and education in a more accessible manner.

## Critical considerations, challenges, and future directions

### Ethical considerations of the use of gen-AI in patient education

Ethical considerations are paramount in any technology-driven initiative, including the use of generative AI for patient education in resource-limited countries. One of the primary concerns is patient privacy,^68^^,^^69^ as the use of AI requires the collection and processing of sensitive patient data. It is essential to ensure that appropriate data protection measures are in place, such as using secure servers, encrypting data, and adhering to international data protection standards,^70^ like the General Data Protection Regulation (GDPR) or the Health Insurance Portability and Accountability Act (HIPAA).

Data security is also a critical consideration,^69^ as the use of AI requires access to large amounts of patient data, including medical histories and imaging results. It is crucial to ensure that appropriate safeguards are in place to prevent data breaches, such as using firewalls and implementing access controls.^71–73^

Another ethical consideration is the role of healthcare professionals in the implementation and use of generative AI.^71^ It is important to recognize that AI technology is not a replacement for healthcare professionals, but rather a tool to enhance their work.^74–76^ Therefore, it is essential to involve healthcare professionals in the development, implementation, and use of AI-based patient education programs to ensure that they align with ethical and clinical standards.

To ensure the responsible and ethical use of generative AI in patient education, best practices should be followed. These may include conducting thorough risk assessments, establishing clear guidelines for data use and sharing, providing transparent communication with patients about the use of AI, and adhering to ethical principles and international data protection standards, such as GDPR and HIPAA.^75^^,^^77^ Additionally, ongoing monitoring and evaluation should be conducted to ensure that the use of generative AI in patient education is effective, safe, and ethical.

### Implementation challenges

Implementing generative AI-based patient education solutions in resource-limited countries is not without challenges. Some of the key obstacles include:

#### Infrastructure

Many resource-limited countries may lack the necessary infrastructure to support AI technology, such as high-speed internet and reliable electricity.^78^ Developing strategies to address these infrastructure challenges will be crucial for the successful implementation of AI-based patient education initiatives.^79^

#### Internet connectivity

Limited or inconsistent internet access can hinder the adoption of AI-based educational tools. Ensuring widespread and affordable internet access is crucial for enabling the use of these technologies in resource-limited settings.^78^^,^^80^^,^^81^

#### Cost

Implementing AI solutions may require significant financial investment, which may be challenging for resource-limited countries. Exploring cost-effective AI models and identifying sources of funding or partnerships will be critical for successful implementation.^78^^,^^81^^,^^82^

#### Technical expertise

A lack of local technical expertise in AI and healthcare can hinder the development and deployment of AI-based patient education programs.^83^^,^^84^ Developing local capacity through training and education is essential for addressing this challenge.

#### Clinical validation

Validating the clinical efficacy and accuracy of AI-generated patient education materials is paramount. Ensuring that AI outputs align with established medical standards and guidelines is necessary for patient safety and trust.^85^

#### Complexity of medical information

Medical information can be complex and nuanced, requiring careful translation into patient-friendly educational content. AI algorithms must effectively distil and communicate this information to ensure patient comprehension.

#### Integration with healthcare systems

Seamless integration of AI-based patient education tools with existing healthcare systems is essential for widespread adoption and effectiveness. Compatibility with electronic health records and clinical workflows must be considered during implementation.

### Limitations

#### Data quality and bias

AI algorithms rely on large datasets for training and generating accurate information. The quality of these datasets is critical for the performance of AI-based patient education tools.^69^^,^^86^

However, in resource-limited countries, data quality may be compromised due to factors such as incomplete records or inconsistent data collection methods.^87^ Furthermore, biases in training data can lead to biased AI outputs, which may adversely affect patient education.^87^^,^^88^ These biases can arise from low sampling of the underrepresented populations or the skewed data, which can result in AI algorithms reinforcing existing stereotypes or perpetuating inequalities.^89^^,^^90^ To mitigate these biases, it is essential to ensure that AI algorithms are trained on diverse and representative datasets that account for the full spectrum of patient demographics, languages, and cultural contexts.

#### Cultural and linguistic diversity

AI algorithms may struggle to adapt to the vast cultural and linguistic diversity in resource-limited countries. This can make it difficult to develop AI-based patient education tools that cater to the unique needs and preferences of different communities.^87^ Ensuring that AI algorithms can handle diverse languages and cultural contexts is crucial for effective patient education in these settings.

#### Trust and acceptance

Patients in resource-limited countries may be unfamiliar with AI technology, leading to mistrust or reluctance to engage with AI-based patient education tools. Building trust and acceptance among patients and healthcare professionals is essential for the successful adoption of AI in patient education.^91^

#### Ethical and legal issues

As mentioned earlier, ethical and legal issues, such as data privacy and security, need to be addressed when implementing AI in patient education. Failure to consider these aspects can lead to unintended consequences and limit the effectiveness of AI-based patient education initiatives. Legal issues related to AI in patient education may include intellectual property rights, liability for incorrect or harmful information generated by AI algorithms, and regulatory compliance with local and international laws governing healthcare and data protection. Ensuring that AI-based patient education tools adhere to relevant legislation and regulations is crucial to avoid legal disputes and protect the rights and interests of patients, healthcare providers, and AI developers.^33^ Additionally, clear guidelines and legal frameworks need to be established to govern the development, use, and evaluation of AI-based patient education initiatives, with particular attention given to addressing the unique challenges faced in resource-limited countries.

### Future directions

The future of AI in patient education, particularly in the context of cardiovascular imaging and resource-limited countries, holds tremendous potential. Some possible future directions include:

- Integration of generative AI with mobile health applications: Combining AI with mHealth apps can provide more accessible and personalized patient education, reaching a wider audience and empowering patients to take a more active role in managing their cardiovascular health.
- Expansion to other healthcare domains: Lessons learned from generative AI-based patient education initiatives in cardiovascular imaging can be applied to other areas of healthcare, such as diabetes management, mental health, and maternal care, to improve patient education and health outcomes in resource-limited countries.
- As GPT-4 and other LLMs continue to evolve, they may further improve patient education and care and can lead to new horizons as more and more applications can be born out of such technologies.
- Advances in NLP, data security, and machine learning algorithms could lead to even more personalized, accurate, and culturally sensitive educational content.

## Conclusion

Through continued research, innovation, and collaboration, generative AI has the potential to revolutionize patient education, creating a more informed and empowered patient population capable of making better decisions regarding their cardiovascular health in resource-limited settings. Cardiovascular diseases are a leading global cause of mortality, necessitating effective patient education for better clinical outcomes. Generative AI offers great potential to enhance patient education regarding cardiovascular imaging in resource-limited countries. By enabling multimedia content generation, personalization, simulations, predictive analytics, and more, generative AI can improve education accessibility and quality, facilitating earlier disease detection and treatment. Ethical considerations, including patient privacy and data security, and the involvement of healthcare professionals in delivering accurate information, require careful attention during implementation. Addressing limitations such as data quality, bias, cultural diversity, trust, and ethical and legal issues is essential for successful AI adoption. Future research should focus on optimizing AI applications through enhanced NLP and adaptive learning algorithms, harnessing the power of chatbots and virtual assistants for real-time support. Through continued research, innovation, and collaboration, generative AI has the potential to revolutionize patient education, creating a more informed and empowered patient population capable of making better decisions regarding their cardiovascular health in resource-limited settings.
